# Plant volatile organic compounds attractive to *Lygus pratensis*

**DOI:** 10.1515/biol-2022-0038

**Published:** 2022-04-11

**Authors:** Hongzu Feng, Changqing Gou, Dilinuer Aimaiti, Peng Sun, Lan Wang, Haiting Hao

**Affiliations:** Key Laboratory of Integrated Pest Management (IPM) of Xinjiang Production and Construction Corps in Southern Xinjiang, The National and Local Joint Engineering Laboratory of High Efficiency and Superior – Quality Cultivation and Fruit Deep Processing Technology of Characteristic Fruit Trees in Southern Xinjiang, Scientific Observing and Experimental Station of Crop Pests in Alar, Tarim University, Alar, Xinjiang Province 843300, China; Natural Resources Bureau of Qitai County, Changji 831800, China; China Cotton Group, Beijing, 100000, China

**Keywords:** electroantennogram, tendency behavior, filed trapping efficiency, attractant, *Lygus pratensis*

## Abstract

*Lygus pratensis*, an important agricultural pest, is seriously detrimental to cotton in China. For the research and development of attractants, the present study screened and identified plant volatiles with activity against the pest. Out of the total 20 volatiles identified from seven hosts, 16 volatiles were selected and evaluated. Electrophysiological test results revealed the highest electroantennogram values of heptacosane, heptadecane, decanal, (*E*)-4-hexen-1-ol, dodecane, β-pinene, and *cis*-3-hexenyl isovalerate on adult insects. A significant difference in the behavior of female adults (*P* < 0.01) was noted in the trend behavioral tests when the concentration of heptacosane, nonadecane, heptadecane, decanal, 3-hexen-1-ol, and dodecane was 10^−3^ (V/V), and that of β-pinene was 10^−4^ (V/V). The field trapping test confirmed a significant difference in the trapping effect of heptadecane at 10^−2^ (V/V) and 10^−3^ (V/V), decanal at 10^−1^ (V/V) and 10^−3^ (V/V), β-pinene at 10^−2^ (V/V), and dodecane at 10^−4^ (V/V) compared to that of other volatiles (*P* < 0.05). These findings suggested the strong attractant effect of heptadecane, dodecane, decanal, and β-pinene on adults, indicating their potential application as effective attractants for the ecological control of *L. pratensis*.

## 1 Introduction

*Lygus pratensis* (Hemiptera: Miridae) is an agricultural pest known to cause damage to various crops, including corn, wheat, cotton, beans, and vegetables. Xinjiang is a large cotton-growing province in China. With the gradual increase in the pest population, it has become the predominant pest in the cotton field [1]. This pest leads to the falling off of a large number of cotton bolls, thereby causing severe damage to cotton cultivation. Moreover, it adversely affects various other important crops such as cereals, vegetables, and fruits [2]. However, chemical pesticides, mainly used to control the bug, negatively affect the environment and human and animal health and indulge in the emergence of drug resistance [3]. This necessitates the development of novel techniques and methods to control the bug. Plant volatiles are secondary metabolites of plants with complex composition and low concentration, released from the above-ground parts of plants. Being important chemical information links between insects and plants, these compounds play a crucial role in insect feeding and oviposition [4,5,6,7,8,9]. With the rapid advancement of behavioral measurement, chemical analysis, and electrophysiological techniques, the interaction between plant volatile organic compounds (VOCs) and herbivorous insects has become a research hotspot [10,11,12,13,14]. Studies have highlighted the relevance of plant VOCs in the life activities of herbivorous insects, such as finding a host, supplementing nutrition, and mating and oviposition [10,11,12,13,14,15]. *Lygus rugulipennis* adults exhibit a directional selection behavior for the odor of flowering sunflower or lucerne. Research claims an obvious tropism effect on *L. rugulipennis* adults attributed to the plant VOCs, phenylacetaldehyde, and monoterpenes, naturally released by these flowers [16,17,18]. *cis*-3-Hexen-1-ol reported a significant attractant effect on the orientation behaviors of both male and female insects (*Phthorimaea operculella*). A previous study elucidated that only female insects were attracted by nonanal and decanal, whereas octanal exerted a repellent effect on male insects [19]. Insect dose responses to plant VOCs can reflect the range of concentrations over which herbivore or parasitoid attraction or repellence may occur [20,21,22]. Benzyl acetate, methyl salicylate, and β-caryophyllene at concentrations of 10, 100, and 1,000 ng/min, respectively, were known to repel both *Gastrophysa viridula* and *Gastrophysa polygoni* leaf beetles. Neither sex of either species, except for *G. viridula* females, demonstrated significant dose responses at a concentration of 1 ng/min [23]. Following *L. rugulipennis* feeding, quinoa plants (*Chenopodium quinoa*) manifested much larger VOC emission compared with that after saponin applications and control. Plant volatiles play a vital role in the “push–pull” strategy. On the one hand, the repellents enable the prevention of the damage caused by the pests to crops, and the luring stimulants are used to lure pests to the established area for centralized elimination; on the other hand, attractants help to trap natural enemies and reduce the density of field pest populations [24]. Further studies should explore the mechanism to stimulate the role of plant volatiles in the “push–pull” strategy to control pests. To screen out effective attractants, we estimated the electroantennogram (EAG) value and trend behavior of 16 plant VOCs and examined the attractant effect of each VOC to lay a theoretical foundation for the development of safe and efficient plant attractants.

## 2 Materials and methods

### 2.1 Test plants

Based on our previous research findings [25,26], seven preferred hosts of the pest, namely *Kochia prostrata*, *Chenopodium glaucum*, *Brassica oleracea*, *Brassica campestris*, *Convolvulus arvensis*, *Chenopodium serotinum*, and *Lycopersicon esculentum*, were selected as the test plants for the present study.

### 2.2 Test insects

After collecting the adult *L. pratensis* from the weeds growing around the Agricultural Science and Technology Industrial Park of the 12th Regiment of Xinjiang First Division, the insects were transported to the laboratory and placed in a 50 cm × 50 cm × 50 cm self-made insect net. The insects were raised with fresh *Phaseolus vulgaris.*

### 2.3 Collection and testing of plant VOCs

#### 2.3.1 Collection of host plant VOCs through solid-phase microextraction

Fresh *in vitro* plants were collected prior to the experiment. The wounds were wrapped with a cotton ball soaked in water to prevent dispersion of volatiles, and the plants were quickly brought back to the laboratory. A 500-mL jar sealed with a silicone stopper was used to place the plant. The extraction head was placed in the gas chromatography (GC) inlet at 250°C and activated for 1 h. The solid-phase microextraction (SPME) handle was fixed with an iron stand. To extract the VOC, the extraction head was slowly pushed out and inserted into the wide mouth bottle. The extraction time was 40 min.

#### 2.3.2 Identification and analysis of host plant VOCs

The experiment was conducted in the Biological Testing Centre of Tarim University. For *K. scoparia*, *C. sylvestris*, *C. sylvestris*, *C. vulgare*, and *L. esculentum,* the extraction head, collecting the plant VOCs, was directly subjected to thermal analysis in the GC inlet at 250°C. The analysis time was 3 min. The gas chromatography-mass spectrometer (GC-MS) working parameters were set following the recommendations of Wu [27]. The chromatographic column was an 5% phenylmethylsiloxane MS column (HP-5MS) capillary column (30 m × 0.25 mm ID; 0.25-µm film thickness), with no split injection; the column temperature was programmed to increase as follows: 40°C (2 min) and 6°C/min to 220°C (2 min). The carrier gas was high-purity (99.999%) helium, with the column gas flow rate adjusted to 1.0 mL/min. The temperature of the GC-MS interface was set at 280°C. Electron ionization (EI) served as the ion source, with the ionization energy being 70 eV and the mode being the full-scan mode.

The extraction head for *B. campestris* and *B. oleracea* collected the host plant volatiles and was directly subjected to thermal analysis in the GC inlet at 250°C, with 5 min analysis time. GC-MS working conditions were in accordance with the method detailed by Dai et al. [28]. An HP-5MS capillary column served as the chromatographic column, the temperature of the detection chamber was set at 250°C, and the sample was split free; the column temperature was programmed to increase as follows: 50°C (2 min), 2°C/min to 120°C, 10°C/min to 160°C, and 20°C/min to 220°C (5 min). High-purity (99.999%) helium was used as the carrier gas, and the column gas flow rate was adjusted to 1.0 mL/min. The temperature of the GC-MS interface was 280°C. EI served as the ion source, the scanning range was 30–500 *m*/*z*, the temperatures of the ion source and the quadrupoles were 250 and 150°C, respectively, and the scanning frequency was 5 times/s.

The NIST2005 spectrogram database and reference documents were nurtured to assess the collected VOCs qualitatively. The area normalization method was adopted to determine the relative content of each component. Some of the identified compounds were common to many host plants, while other compounds were unique to each host plant. This experiment screened 16 VOCs common to many host plants (more than 3 hosts) and unique to each host plant. These standard compounds were then purchased (Appendix).

### 2.4 EAG measurement

The EAG, manufactured by Syntech company (The Netherlands), comprised an IDAC-2 dual-channel USB interface, signal acquisition controller, probe signal amplifier, and MP-15 micro-operation platform. The company also provided the software required for EAG determination. First, 6- to 10-day-old female *L. pratensis*, collected using a finger tube, were placed on ice for anesthetization of the insects. Subsequently, the antennae were cut off, connecting the end of the incision with a reference electrode (the capillary contained 0.9% NaCl solution). The top of the antennae was connected with the recording electrode and the two electrodes were attached through a silver wire. Paraffin oil and *cis*-3-hexen-1-ol served as the blank control and internal control, respectively. The internal control and standard odor samples (Table A1) were prepared at a concentration of 100 µL/mL, and the test dose was 10 µL. Configured 2 µL reagent, extracted using a microsampler, was evenly dropped on a 2 cm × 0.5 cm clean filter paper; the filter paper was then placed into a Pasteur pipette, and the instrument was debugged. The response value of the antenna potential was recorded only after the stabilization of the EAG signal. For each head of the insect, the control stimulation test was performed, followed by the reference stimulation test. The remaining standard compounds were then screened randomly, and finally, the reference and control stimulations were tested separately. The stimulation time was 0.5 s, the stimulation interval was 30 s, and the experiment was repeated six times. An IDAC-2 signal collector mediated the connection between the EAG instrument and the computer. The antenna was approximately 1 mm away from the orifice of the air mixing tube, and air at a flow rate of 150 mL/min was passed through the activated carbon and humidifying bottle that entered the airflow mixing pipe. Subsequently, the stimulus source was blown toward the antennae, and the stimulating air flow rate was 20 mL/min. During the test, the ambient temperature and humidity were adjusted to 25 ± 2°C and 60–70%, respectively. The relative value (*R*) of the EAG reaction of the volatiles was computed using the following formula: *R* = (volatile reaction value − control reaction value)/(reference reaction value − control reaction value); *R* represents the relative value of the EAG reaction of volatiles. The absolute value difference of EAG response to candidate volatiles was compared with Duncan’s new complex range method in SPSS 22.0 statistical software. Student’s *t-*test was exploited to explore the significance of EAG response between female and male insects toward identical volatiles.

### 2.5 Determination of olfactory tendency behavior

**Figure A1:**
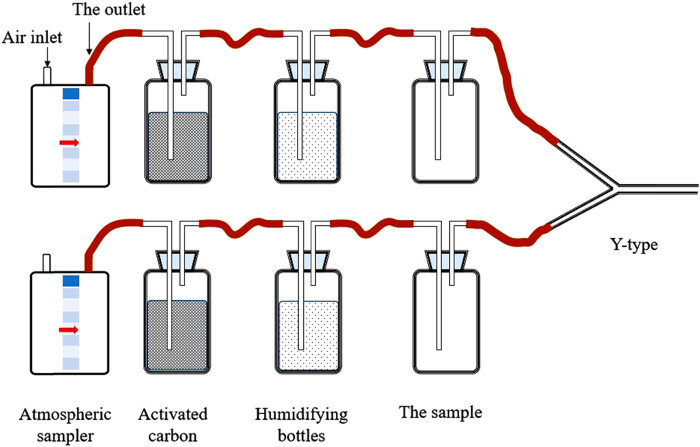
Installation diagram of Y-tube olfactmeter.

The olfactory tendency behavior was ascertained with the method described by Pan et al. [29], with slight modifications. The tropism response of the insect toward the VOCs was determined by a Y-type olfactory instrument (Figure A1). The Y-type olfactory instrument was composed of colorless transparent glass with an inner diameter of 3 cm, and its base and both arms were 15 cm long. The angle of the two arms was 60°. The base was connected with the release tube of the adult insect, the arms were plugged tightly with corks, and the odor source was connected with a glass tube.

Liquid paraffin (10 µL) containing a single-component pure product was dripped onto the half-folded filter paper strip (0.5 cm × 5 cm). After 30 s, the filter paper strip placed into an Erlenmeyer flask served as the odor source. Liquid paraffin was used as the solvent and control. The Quality Control-1B (QC-1B) air sampler was used as the airflow power system, and the odor source was connected to a vacuum pump. Filtered by activated carbon and humidified with distilled water, the air was then allowed to enter the odor source. The gas flow rate was set at 500 mL/min. An adult insect was placed at the base of the Y-shaped tube. The stop clock was started when the insect reached half the length of the base tube. The behavioral response was observed for 5 min. The evaluation criteria were as follows: if the insect crossed 3 cm of a certain arm and remained in this area for more than 5 s, the test insect was considered to select the odor source connected to the arm. If the adult failed to react within 5 min of being placed in the tube, the test insect was recorded as being nonresponsive. Each adult was screened only once. For each test, the direction of the left and right arms of the Y-tube was exchanged once. The Y-tube was replaced while testing the 10th insect. Each group comprised 60 male and 60 female insects. The insects were starved for 5 h prior to the experiment. The test was performed at 26 ± 1°C. After the daily test, acetone was used to clean the Y-tube, gas cylinder, and connecting hose. The *χ* ^2^ test was used to evaluate whether the choice between two odor sources for the insect demonstrates a theoretical distribution. The H_0_ value was assumed to be 50:50. Thereafter, the *χ* ^2^ value and corresponding *P*-value were computed.

### 2.6 Field trapping effect of volatile substances

According to our previous research results [30], a white armyworm board (length 24 cm and width 40 cm) was selected. The front and back sides of the insect board were covered with sticky shellac. The experiment site was the garden nursery base of the Tarim University. To avoid the effect of human interference on the test results, operations such as chemical pesticide spraying, agricultural operations, and field surveys not related to the test were canceled during the test period. Before the field test, long bamboo poles were inserted into the soil and fixed in a vertical position. With thin iron wires, the sticky boards were then fixed on the bamboo poles. It was ensured that the bottom was slightly higher than the top of the weeds. The distance between two adjacent bamboo poles was 15 m. The sticky surface of the insect board was hung to the south. In the center of the sticky plate, 1 mL of active substances diluted with lanolin paste were hung as the control. Placing these substances in a 2 mL centrifuge tube, the orifice of the tube was inclined slightly downwards. After three days, the insects caught on the sticky insect board were identified, and the number of insects was counted. The experiment with each active substance analog was repeated six times. Finally, Duncan’s new multiple range method was applied to verify the significance of the difference.

## 3 Results

### 3.1 Identification and analysis of plant volatiles

GC-MS combined with the computer retrieval technology was utilized for the isolation and identification of the compounds. Several VOCs were isolated from seven host plant species (Table 1). The present study identified a total of 16 compounds. Most of these compounds were extracted from *C. glaucum, B. oleracea*, and *L. esculentum* (with four species each), followed by *C. serotinum* and *B. campestris* (with three species each), and *K. prostrata* and *C. arvensis* (with two species each). Though some of these compounds were common to many host plants, others were unique to each host plant. The alkane and ester groups (10 types) were found to be the most frequently detected components among the identified compounds, followed by alcohols (two types) and terpenes, aldehydes, ketones, and amides (with one type for each). Among the seven host plants, dodecane belonged to *C. glaucum*, *B. oleracea*, *C. arvensis*, and *B. campestris*. Tetratetracontane was obtained from *C. glaucum*, *B. oleracea*, and *L. esculentum*. The GC-MS area normalization method calculated the percentage content of each component. Results revealed <3% relative content of the identified compounds in the respective host plants, which was considered as the trace level (Table 1).

**Table 1:** The name and content of volatiles of different host plants

Volatile compound	Retention time/min	Relative content (%)						
		A	B	C	D	E	F	G
4-Hexen-1-ol, (*E*)-	6.30	—	—	—	—	—	—	0.44
3-Hexen-1-ol	7.10	—	0.38	—	—	—	—	—
1-Hexanol, 2-ethyl-	11.97	0.09	—	—	—	—	0.2	—
*cis*-3-Hexenyl butyrate	12.85	—	—	—	—	—	—	1.40
β-Pinene	13.52	—	—	1.01	—	—	—	—
Nonanal	13.95	0.04	0.04	—	—	—	—	—
Crystal Violet Lactone	16.83	0.26	—	—	—	0.19	—	—
*cis*-3-Hexenyl isovalerate	17.22	—	—	—	0.07	—	—	—
Heptadecane	17.85	—	—	—	—	—	0.08	—
β-ionone	20.58	—	—	—	—	—	—	0.08
Nonadecane	20.63	0.29	—	—	—	—	—	—
1-caryophyllene	21.20	—	0.13	—	0.49	—	—	—
Dodecane	22.30	—	0.22	1.64	0.03	0.43	—	—
Pentadecane	22.83	0.14	—	—	—	—	—	—
Heptacosane	25.57	—	0.84	—	0.41	—	0.20	—
Decanal	27.25	—	—	1.32	—	—	—	—
Spironolactone	28.93	—	—	—	—	0.16	—	—
Octacosane	34.42	—	—	—	—	—	0.14	—
Tetratetracontane	41.48	—	0.15	0.69	—	—	—	0.09
Octadecanamide	46.13	—	—	—	—	2.04	—	—

A: *K. prostrata*; B: *C. glaucum*; C: *B. oleracea*; D: *C. arvensis*; E: *B. campestris*; F: *C. serotinum*; G: *L. esculentum*.

### 3.2 The EAG response of the insect to the host plant volatile odor standard sample

**Figure 1:**
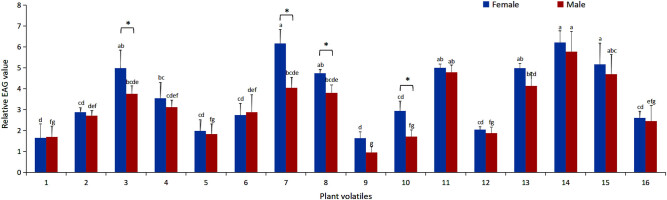
EAG responses of *L. pratensis* to host plants volatiles: (1) Tetratetrac, (2) Octacosane, (3) Heptacosane, (4) Nonadecane, (5) Heptacosane, (6) Spironolac tone, (7) Heptadecane, (8) Decanal, (9) β-ionone, (10) *cis*-3-Hexenyl butyrate, (11) (*E*)-4-Hexen-1-ol, (12) 3-Hexen-1-ol, (13) Dodecane, (14) β-Pinene, (15) *cis*-3-Hexenyl isovalerate, and (16) Pentadecane. Note: Different letters on the column denote significant differences in the relative value of EAG responses of *L. pratensis* to various plant volatiles of the same type at a *p*-value <0.05 based on Duncan’s new multiple range test. * indicates significant difference between female and male insects at a *p*-value <0.05, determined through Student’s *t*-test.

Significant differences (*P* < 0.05) were prominent in the EAG responses of female and male adults to 16 plant volatiles. Compared to the male adults, the female adults manifested higher EAG responses to the same plant volatiles; however, responses to only heptadecane, decanal, and *cis*-3-hexenyl butyrate demonstrated a significant difference between the male and female insects (*P* < 0.05) (Figure 1).

### 3.3 Behavioral response of *L. pratensis* to host plant volatile odor standards

**Figure 2:**
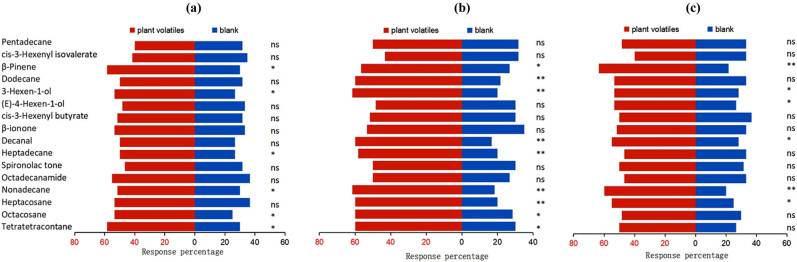
Response of adult *L. pratensis* females to different host plants volatiles. (a) The behavioral response at the concentration of 10^–2^ (V/V). (b) The behavioral response at the concentration of 10^–3^ (V/V). (c) The behavioral response at the concentration of 10^–4^ (V/V). ns represents no significant difference at the 0.05 level; * indicates a significant difference at the 0.05 level; ** denotes an extremely significant difference at the 0.01 level.

**Figure 3:**
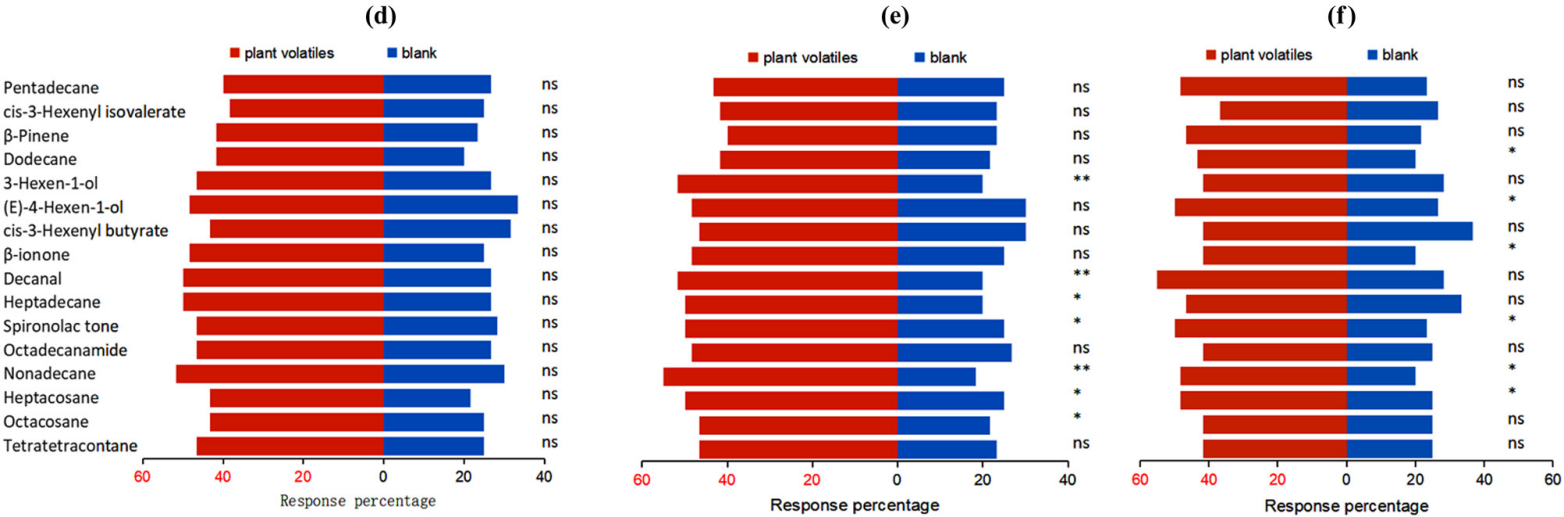
Response of adult *L. pratensis* males to different host plants volatiles (d) the behavior response at the concentration of 10^–2^ (V/V); (e) the behavior response at the concentration of 10^–3^ (V/V); (f) the behavior response at the concentration of 10^–4^ (V/V). ns indicates no significant difference at the 0.05 level; * implies a significant difference at the 0.05 level; ** designates an extremely significant difference at the 0.01 level.

Higher behavioral responses of the female insects than that of the male insects were documented at 10^–2^, 10^–3^, and 10^–4^ (V/V) of the 16 compounds (Figures 2 and 3). At 10^–3^ (V/V) of the same compound, the female and male adults exhibited the highest behavioral response. A significant difference was observed by the female adults in response to heptacosane, nonadecane, heptadecane, decanal, 3-hexen-1-ol, and dodecane at 10^–3^ (V/V) and β-pinene at 10^–4^ (V/V) (*P <* 0.01). Male adults witnessed significant attraction toward nonadecane, decanal, and 3-hexen-1-ol at 10^–3^ (V/V) (*P <* 0.01).

### 3.4 The field trapping effect of plant volatiles on *L. pratensis*

To rule out the interference of color of the sticky insect board on the field trapping effect of plant volatiles, the white sticky insect board was selected. Results confirmed that the white sticky insect board reflected the worst trapping effect. Variation in the concentrations of plant volatiles altered the attraction effects on male and female adults, as evident in Table 2. In comparison, the best effects were manifest by β-pinene at a concentration of 10^–2^ (V/V), heptadecane and decanal at a concentration of 10^–3^ (V/V), and dodecane at a concentration of 10^–4^ (V/V) and significantly differed from those of other volatiles (*P <* 0.05) (Table 2).

**Table 2:** Field trapping effect of plant volatile substances on adult male and female *L. pratensis*

Plant volatile	Female (head/board/day)				Male (head/board/day)			
	10^-1^ (V/V)	10^-2^ (V/V)	10^-3^ (V/V)	10^-4^ (V/V)	10^-1^ (V/V)	10^-2^ (V/V)	10^-3^ (V/V)	10^-4^ (V/V)
Tetratetracontane	1.6 ± 0.4abc	2.4 ± 0.6ab	2.6 ± 0.6bcde	1.8 ± 0.4abc	1.2 ± 0.4abcd	1 ± 0.4b	2.2 ± 0.5abcd	1.4 ± 0.3abc
Octacosane	2.2 ± 0.7abc	1 ± 0.0bc	2.4 ± 0.8cde	1.6 ± 0.3abc	1.2 ± 0.2abcd	1 ± 0.5b	2 ± 0.4abcd	1.4 ± 0.3abc
Heptacosane	2.2 ± 0.7abc	1.4 ± 0.3bc	2 ± 0.5def	2.2 ± 0.7ab	2 ± 0.6ab	1.4 ± 0.6ab	1.6 ± 0.6bcd	1.6 ± 0.6abc
Octadecanamide	2 ± 0.4abc	2 ± 0.8ab	2.4 ± 0.3cde	1.4 ± 0.6abc	1.8 ± 0.4abc	1.8 ± 0.4ab	2 ± 0.4abcd	1.4 ± 0.4abc
Heptacosane	0.8 ± 0.4bc	1.6 ± 0.6bc	1.6 ± 0.6def	2 ± 0.0ab	0.6 ± 0.4 cd	1.4 ± 0.8ab	1.8 ± 0.7abcd	1.4 ± 0.4abc
Spironolactone	1.2 ± 0.2bc	1.4 ± 0.7bc	1 ± 0.0ef	2 ± 0.4ab	1.2 ± 0.4abcd	1.2 ± 0.4bc	1.8 ± 0.7abcd	1.8 ± 0.2abc
Heptadecane	2.2 ± 0.4abc	3.4 ± 0.8a	4.4 ± 0.6a	1.4 ± 0.4abc	1.6 ± 0.6abcd	2.4 ± 0.8ab	3.4 ± 0.6ab	1.2 ± 0.4abc
Decanal	3 ± 0.8a	1.2 ± 0.4bc	4.2 ± 0.7a	2.6 ± 0.7ab	2.4 ± 0.86a	1 ± 0.6b	3.6 ± 0.6a	1.4 ± 0.6abc
β-ionone	1 ± 0.6bc	1.4 ± 0.4bc	1.8 ± 0.2def	1.4 ± 0.8abc	1 ± 0.4bcd	1 ± 0.6b	1.4 ± 0.7 cd	1.2 ± 0.7abc
*cis*-3-Hexenyl butyrate	0.8 ± 0.4bc	1.2 ± 0.2bc	2.2 ± 0.5cdef	2.4 ± 0.6ab	0.6 ± 0.3 cd	1.2 ± 0.8b	1.6 ± 0.4bcd	2.2 ± 0.7ab
(E)-4-Hexen-1-ol	1 ± 0.4bc	1.8 ± 0.4bc	1.6 ± 0.7def	2 ± 0.4ab	0.8 ± 0.4bcd	1.2 ± 0.2b	1.4 ± 0.6 cd	2 ± 0.4abc
3-Hexen-1-ol	1 ± 0.6bc	1.8 ± 0.4bc	1.8 ± 0.5def	1 ± 0.4bc	1 ± 0.4bcd	1.8 ± 0.7ab	1.6 ± 0.6bcd	0.8 ± 0.4bc
Dodecane	2.4 ± 1.0ab	2.6 ± 0.4ab	1.4 ± 0.4def	3 ± 0.8a	1.2 ± 0.4abcd	2.6 ± 1.5ab	2.6 ± 1.2abc	2.8 ± 1.0a
β-Pinene	1 ± 0.5bc	3.4 ± 0.4a	4 ± 0.8ab	2.4 ± 0.6ab	0.8 ± 0.4bcd	3.2 ± 1.0a	3.2 ± 1.0abc	2.2 ± 0.8ab
*cis*-3-Hexenyl isovalerate	1 ± 0.0bc	1.8 ± 0.7bbc	3 ± 0.5abcd	2.2 ± 0.7ab	1 ± 0.4bcd	1.2 ± 0.2b	2.6 ± 0.6abc	1.2 ± 0.5abc
Pentadecane	0.8 ± 0.4bc	2. 4 ± 0.8ab	3.6 ± 0.6abc	2.6 ± 0.3ab	0.6 ± 0.4 cd	1.8 ± 0.7ab	2.4 ± 0.6abc	1.4 ± 0.6abc
Control	0.6 ± 0.3c	0.4 ± 0.3c	0.6 ± 0.3 f	0.4 ± 0.3c	0.4 ± 0.3d	0.8 ± 0.2b	0.4 ± 0.3d	0.4 ± 0.3c

Note: Data are presented as mean ± SE. Different letters are significantly different between different treatments (Duncan’s new multiple range test, *P* < 0.05).

## 4 Discussion

To extract the volatiles from seven host plant species, the present study used the SPME method. The GC-MS analysis screened a total of 20 VOCs. Based on the existing literature, 16 compounds with potential activity were selected for EAG reaction, the olfactory behavior, and field trapping tests. The female adults of *L. pratensis* revealed a strong EAG response and trend behavioral response to heptacosane, heptadecane, decanal, dodecane, and β-pinene. Nonetheless, inconsistency was observed among the EAG response to (*E*)-4-hexen-1-ol and 3-hexen-1-ol with the behavioral response results; this phenomenon is often witnessed among herbivorous insects [31]. Williams et al. [32] reported that the female and male adults of *L. hesperus* exhibited a low EAG response to β-pinene but an obvious behavioral response, as the electrophysiological response to *n*-hexanol was high but no obvious behavioral tendency was recorded in their study.

Relative to the male adults, a higher attractant effect of the 16 VOCs was noted on female adults. This difference could be attributed to the roles of male and female insects in finding hosts, reproducing offspring, or other aspects. Moreover, the persistence of gender-based differences in the antenna receptors of the insect may contribute to the variations in the perception of smell between male and female insects.

Field trapping experiments reported superior trapping effects on *L. pratensis* when the concentration of β-pinene was 10^–2^ (V/V), that of heptadecane and decanal was 10^–3^ (V/V), and that of dodecane was 10^–4^ (V/V). The results of the field experiment were in agreement with that of the indoor experiment. On the one hand, the selected volatiles illustrated an important reference value for the development of high-efficiency attractants for *L. pratensis*, on the other hand, they also reflected their potential application as components of repellents or attractants. Inevitably, the effects of some plant volatiles differed in indoor and field experiments. For instance, the weak insect-attracting effect of octadecanamide, spironolactone, *cis*-3-hexenyl isovalerate, and pentadecane under indoor conditions was in contradiction with their significantly higher trapping effect than most other volatiles witnessed in the field. Research has substantiated varying behavioral effects of different host plant volatiles on herbivorous insects. As long-distance attractants, some substances provide long-distance (directional) attraction to insects. Considerable attenuation of the attractant effect of these substances is observed, once an adult enters a certain distance. At this time, other substances in a relatively close range stimulate adults and induce female adults to lay eggs or feed [33,34]. Therefore, we speculate a strong directional attraction to *L. pratensis* contributed by octadecanamide, spironolactone, *cis*-3-hexenyl isovalerate, and pentadecane at a long distance; however, the lure of feeding and laying eggs was found to be weakened at a close range. Conversely, heptadecane, dodecane, decanal, and β-pinene were assumed to impose strong effects on *L. pratensis* feeding and laying eggs at a close range and provide directional attraction at a long distance, although this speculation needs further research and confirmation. The induction of multiple compounds was responsible for the selection and positioning of the host for herbivorous insects. Therefore, the combination of different concentrations of the main volatiles facilitates the selection of a mixture with a strong attractant effect on *L. pratensis*; however, further research in this regard is warranted.

## 5 Conclusions

Thus to summarize, our study explored the attractiveness of plant volatiles to *L. pratensis* with the help of GC-MS, EAG assessment, olfactory behavioral response, and field trapping tests. Our results confirmed the promising effect as an attractant of heptadecane, dodecane, decanal, and β-pinene that can be nurtured for the ecological control of *L. pratensis*. Further elucidation of a mixture with a strong attractant effect on *L. pratensis* by combining the main volatiles at different compositions is highly warranted.

## Appendix

**Table A1:** The odour standard sample and internal control in the experiment

Compound	CAS number	Purity (%)	Producer
3-Hexen-1-ol	544-12-7	98	Sigma-Aldrich
Nonadecane	629-92-5	99	Tianjin Xiensi Biochemical Technology Co., Ltd
Pentadecane	629-62-9	99	Tianjin Xiensi Biochemical Technology Co., Ltd
β-Pinene	127-91-3	>95	Shanghai Jianglai Biotechnology Co., Ltd
*cis*-3-Hexenyl isovalerate	35154-45-1	98	Adamas-bete
Heptadecane	629-78-7	99	Aladdin
β-ionone	14901-07-6	97	Saen Chemical Technology (Shanghai) Co., Ltd
(*E*)-4-Hexen-1-ol	928-92-7	97	Shanghai Bede Pharmaceutical Technology Co., Ltd
Tetratetracontane	7098-22-8	97	FINE
Dodecane	112-40-3	98	FINE
Heptacosane	593-49-7	98	FINE
Octacosane	630-02-4	98	Macklin
*cis*-3-Hexenyl butyrate	16491-36-4	98	Shanghai Yunyuan Reagent Co., Ltd
Decanal	112-31-2	98	Sigma-Aldrich
Spironolactone	1952-1-7	98	FINE
Octadecanamide	124-26-5	98	FINE
*cis*-3-Hexen-1-ol	928-96-1	98	Wuhan Pulov Biotechnology Co., Ltd
